# Pilot Study on the Use of Rheology and Low Field Nmr to Characterize the Liver of Obese Patients Undergoing Metabolic and Bariatric Surgery

**DOI:** 10.3390/ijms27094040

**Published:** 2026-04-30

**Authors:** Alice Biasin, Silvia Palmisano, Michela Abrami, Deborah Bonazza, Fabrizio Zanconati, Domenico Tierno, Federica Tonon, Nhung Hai Truong, Thanh Dang Minh, Ralf Weiskirchen, Fulvia Felluga, Bruna Scaggiante, Mario Grassi, Gabriele Grassi

**Affiliations:** 1 Department of Medical, Surgical and Health Sciences, Cattinara University Hospital, Trieste University, Strada di Fiume 447, I-34149 Trieste, Italy; alice.biasin@phd.units.it (A.B.); spalmisano@units.it (S.P.); fabrizio.zanconati@asugi.sanita.fvg.it (F.Z.); domenico.tierno@units.it (D.T.); federica.tonon@asugi.sanita.fvg.it (F.T.); 2 Surgical Division, Cattinara Hospital, I-34149 Trieste, Italy; 3 Department of Engineering and Architecture, Trieste University, Via Valerio 6, I-34127 Trieste, Italy; michela.abrami@dia.units.it; 4 Unit of Surgical Pathology, Cattinara Hospital, Azienda Sanitaria Universitaria Giuliana Isontina (ASUGI), I-34149 Trieste, Italy; deborah.bonazza@asugi.sanita.fvg.it; 5 Laboratory of Regenerative Biomedicine, University of Science-VNUHCM, Ho Chi Minh City 700000, Vietnam; thnhung@hcmus.edu.vn (N.H.T.); dmthanh@hcmus.edu.vn (T.D.M.); 6 Faculty of Biology and Biotechnology, Viet Nam National University, Ho Chi Minh City 700000, Vietnam; 7 Institute of Molecular Pathobiochemistry, Experimental Gene Therapy and Clinical Chemistry (IFMPEGKC), RWTH University Hospital Aachen, D-52074 Aachen, Germany; rweiskirchen@ukaachen.de; 8 Department of Chemical and Pharmaceutical Sciences, University of Trieste, I-34127 Trieste, Italy; ffelluga@units.it; 9 Department of Life Sciences, University of Trieste, I-34127 Trieste, Italy; bscaggiante@units.it

**Keywords:** liver fibrosis, rheology, Low Field-Nuclear Magnetic Resonance, iron

## Abstract

*Background and aims*. Liver mechanical properties’ (stiffness/viscoelasticity) evaluation is relevant for diagnosing/monitoring liver fibrosis. Due to limitations of the commonly used elastography, we propose the use of rheology and Low Field-Nuclear Magnetic Resonance (LF-NMR). *Methods.* In 30 liver samples from patients undergoing bariatric surgery and 18 control samples, we evaluated the shear modulus *G*/critical stress τc (elastic properties) and mean complex modulus $G_{a}^{*}$ (elastic/viscous properties) by rheology. LF-NMR was used to measure the spin–spin relaxation time (*T*_2m_), reflecting iron content. The expression of iron-related proteins and of pro-fibrotic proteins were evaluated by qRT-PCR. Tissue histology was also determined. *Results*. $G_{a}^{*}$/*G*/τ_c_ were higher in pathological samples, which also showed increased expression of pro-fibrotic proteins. Fibrosis determination displayed a correspondence of 4/30 samples for elastography/histology and 17/30 for rheology/histology. *T*_2m_ was significantly lower in pathological livers, indicating iron accumulation as confirmed by increased expression of iron-related proteins. *T*_2m_ was more effective than histology in detecting iron. An inverse correlation was observed between *T*_2m_ and $G_{a}^{*}$/*G* showing that iron accumulation is associated with increased liver elasticity/viscoelasticity, i.e., fibrosis. Additionally, an inverse correlation of $G_{a}^{*}$/*G* with transferrin, was observed. *Conclusion.* As our patients mostly have mild liver fibrosis, the combined use of rheology/LF-NMR can effectively detect early changes in liver mechanical properties, aiding in staging and diagnosis of fibrosis.

## 1. Introduction

Several different causes are responsible for the induction of liver fibrosis, which eventually results in impaired liver function [1]. Regardless of the etiology, a key element in liver fibrosis is the excessive production of extracellular matrix (ECM) by hepatic stellate cells, which trans-differentiate into proliferative and migratory myofibroblasts [2]. The newly synthesized ECM is characterized by the over-production of various components, including fibronectin and collagen type I [3,4,5]. Tissue remodeling in liver fibrosis is also marked by increased expression of different matrix metalloproteinases (MMPs), including MMP-2 and MMP-9 [6]. MMPs, capable of degrading various ECM components, are regulated at the transcriptional, post-transcriptional and post-translational levels. They play critical roles in the pathogenesis of liver fibrosis, cirrhosis, and liver cancer. Also contributing to liver fibrosis is the increased production of pro-fibrotic proteins like E2F1 [7], a transcription factor involved in regulating cellular processes like proliferation and apoptosis in various tissues, including the liver [8]. E2F1 is implicated in the development of hepatocellular carcinoma, liver fibrosis and cirrhosis [7,8]. Liver fibrosis is further characterized by the accumulation of iron. Iron excess combines with reactive oxygen species, increasing levels of hydroxyl radicals that lead to the peroxidation of cellular macromolecules [9]. This results in hepatic cellular damage and contributes to impaired liver function.

Tissue remodeling in liver fibrosis results in a progressive change in mechanical properties, including stiffness [10,11] and viscoelasticity [12,13]. Therefore, assessing the mechanical properties of the liver is important for evaluating and monitoring liver fibrosis. This is particularly important in patients with metabolic dysfunction-associated steatotic liver disease (MASLD), which encompasses a spectrum of different liver pathological conditions [14,15]. The primary therapeutic goal in MASLD is based on weight loss. When lifestyle modifications and/or drug treatment cannot achieve this, metabolic and bariatric surgery becomes a recommended strategy. Before bariatric surgery, patients undergo ultrasound elastography to estimate the degree of liver fibrosis. Elastography is a non-invasive method to estimate the mechanical properties of the liver in vivo. This technique determines the relationship between wave propagation and the elastic properties of the tissue [16]. Unfortunately, shear-wave elastography is unable to provide reliable data when the thickness of the abdominal wall exceeds 2.5 cm [17], a fact very common in obese patients.

In this pilot study we investigated the combined use of Low Field Nuclear Magnetic Resonance (LF-NMR) and rheology to assess iron content and changes in mechanical properties in liver samples from patients with obesity undergoing metabolic and bariatric surgery. Our method, conducted directly on liver tissue, overcomes the limitations of elastography. However, it is only applicable to patients from whom a liver sample can be obtained. Using LF-NMR, we measure the spin–spin relaxation time (*T*_2m_) of liver tissue samples. Since *T*_2m_ in the liver is primarily affected by iron levels [18], this measurement can offer valuable insights into liver iron content. LF-NMR is a non-destructive, cost-effective technique that does not require any sample alteration, allowing for subsequent analysis such as rheological analysis. The viscoelastic properties of the liver samples are evaluated using two common rheological tests: stress sweep (SS) and frequency sweep (FS). SS tests help identify the maximum deformation a sample can undergo without affecting its internal micro-nanostructure; FS tests determine the sample’s elastic (G′) and viscous (G″) properties.

Our data suggest that the combined use of rheology and LF-NMR can detect alterations in liver physical properties, which could be valuable for diagnosing and staging liver fibrosis.

## 2. Results

### 2.1. Elastography vs. Histology

In this pilot study, thirty obese patients who underwent metabolic and bariatric surgery were included. The characteristics of the patients are detailed in Table 1 and Appendix A.2. As part of the standard evaluation for liver stiffness, the patients underwent liver shear-wave elastography. Reliable shear-wave elastography data were obtained for only seven patients (underlined in Table 1) with an abdominal wall thickness of ≤2.5 cm. The median stiffness values from elastography were categorized into Kleiner–Brunt fibrosis stages (F0–F4) [19] and then compared to the fibrosis level determined by histological analysis (Table 1 and Appendix A.10, representative imagines). Among these seven patients, a correlation between fibrosis evaluated by elastography and histology was evident for only four of them (patients 2, 5, 15, and 21, indicated with * in Table 1).

**Table 1:** Patient demographics and clinical parameters related to fibrosis, steatosis stages and iron.

Pat. N.	Sex	Age	BMI	Abdomen Wall Thickness (cm)	Fibrosis Stage (Histology)	Steatosis Stage (Histology)	Elastography Median Stiffness (kPa)	Fibrosis Stage (Elastography)	Rheology Elasticity *G* (kPa)	Fibrosis Stage (Rheology)	Iron (Perls Staining)
1	F	38	47.9	3.51	0	1			6.1	0	-
2 *	F	62	36.7	2.20	0	1	5.30	0	7.3	2	-
3	F	45	39.1	-	1A	0			5.8	0	-
$\underset{-}{4}$	M	47	33.3	2.07	1C	1	3.76	0	10.4	3	-
5 *	F	41	38.9	2.29	1C	1	6.37	1	6.2	1	-
6	M	53	44.9	4.87	0	1			10.2	3	-
7	F	61	40.6	3.75	0	1			2.7	0	-
8	F	54	42.2	3.20	1A	3			7.0	1	-
9	F	56	47.1	3.75	1B	1			1.9	0	-
$\underset{-}{10}$	F	54	33.7	1.94	1B	1	4.36	0	9.3	3	-
11	F	61	35.1	-	1B	0			3.3	0	+
12	F	29	47.2	2.80	1A	0			23.4	4	-
13	F	59	36.0	2.62	2	1			5.9	4	-
14	M	56	47.3	3.81	1	1			2.5	0	-
15 *	F	32	39.5	2.56	0	0	5.05	0	6.9	1	-
16	M	51	42.6	3.12	1	1			8.9	2	-
17	M	41	42.6	4.51	1A	3			3.7	0	-
18	M	47	40.0	3.16	1A	0			6.6	1	-
19	M	34	33.1	2.75	0	0			3.7	0	+
20	F	45	41.2	2.94	0	1			4.0	0	-
21 *	F	33	38.0	2.57	0	2	3.83	0	3.7	0	-
22	F	46	45.9	4.14	0	1			3.8	0	-
23	M	59	44.5	2.82	0	1			2.5	0	-
24	F	57	39.4	-	1C	1			2.6	0	-
25	F	63	45.1	5.58	0	1			6.6	1	-
26	F	39	37.8	3.10	1	2			49.4	4	-
27	F	52	45.5	2.52	0	0	6.13	1	14.3	4	-
28	M	46	39.5	3.91	0	1			16.2	4	-
29	M	63	38.7	2.93	0	0			10.7	4	-
30	M	33	40.1	-	1	1			51.1	4	-

Underlined: patients for whom elastography was possible to perform; * patients for whom a correlation between fibrosis evaluated by elastography and histology was evident; Green: patients for whom a concordance was obtained for fibrosis determination using rheology and histology: in this regard, to analyse the relationship between Kleiner–Brunt fibrosis and steatosis stages and the rheological data, the following correlation was utilized [20]: F0: 1–6 kPa, F1(1A, 1B, 1C): 6.1–7 kPa, F2: 7.1–9 kPa, F3: 9.1–10.3 kPa, F4: ≥10.4 kPa. Perls stainig is reported as positive (+) or negative (-).

### 2.2. Rheology

**Figure 1:**
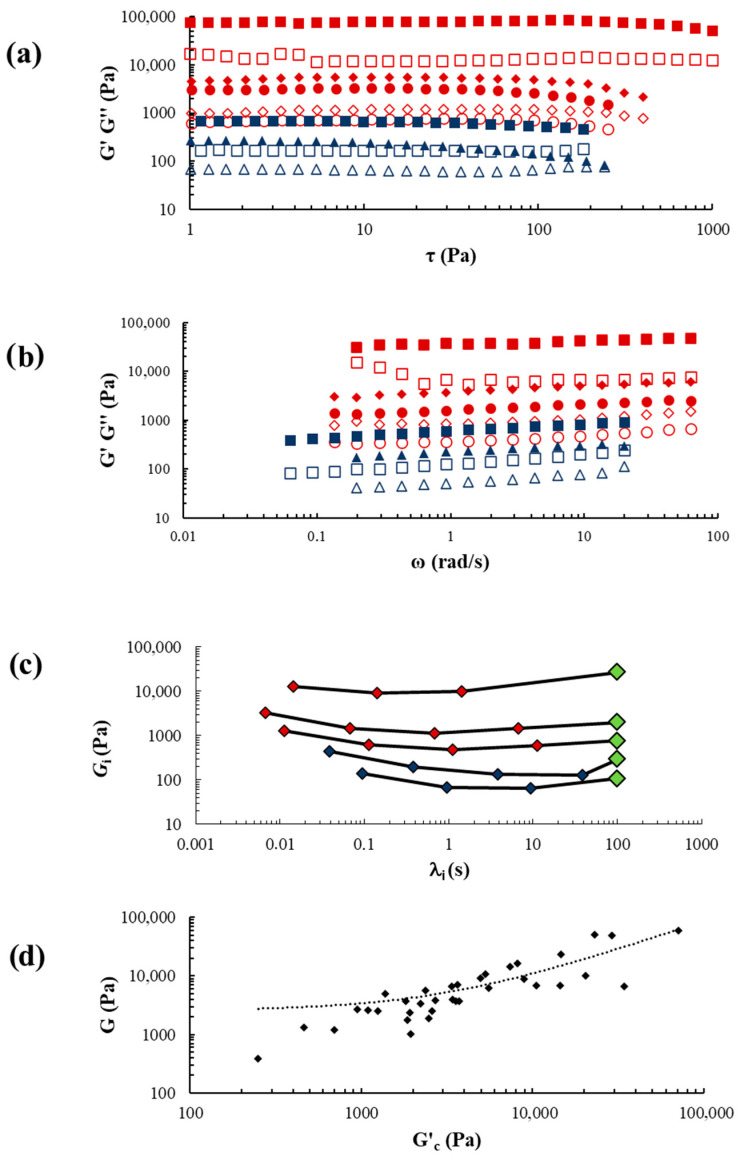
SS, FS, relaxation spectrum and *G*/*G′_c_* in healthy and pathological liver samples. Results of SS (**a**) and FS (**b**) for three representative pathological liver samples (red symbols) and two representative healthy samples (blue symbols). The fully colored symbols represent the elastic modulus (*G′*; *σ_G′_* < 0.1*G′) while the open symbols represent the viscous modulus *G″* (*σ_G__″_* < 0.1*G″). (**c**) The relaxation spectrum for three representative pathological liver samples (red symbols) and two representative healthy samples (blue symbols) is shown. The green rhombuses represent the purely elastic contribution (*G*_E_, appearing in the generalized Maxwell model Equation (1)) to the shear modulus of *G* (*σ_Gi_* < 0.15*G_i_, (*σ*_λ*i*_ < 0.2*λ_i_). (**d**) Correlation between *G* (*σ_G_* < 0.05*G, *σ_G′c_* < 0.3*G′_c_) and *G′_c_* in healthy and pathological liver samples considered together (Spearman correlation r = 0.87 and *p* < 0.0001). *G′*_c_ represents the *G*′ critical value, i.e., the *G*′ value at the end of the sample linear viscoelastic zone (see Equation (5)).

SS (Appendix A.3) and FS (Appendix A.4) tests were conducted on both pathological and healthy liver samples. Figure 1a,b displays the result of the SS and FS tests for representative samples (3 pathological samples, red symbols, patients 10, 13, 19 from Table 1/Appendix A.2, and 2 healthy samples, blue symbols, patients 3, 4 from Appendix A.3, (black numbers), along with the relaxation spectra (Figure 1c) derived from fitting the generalized Maxwell model (Equations (1) and (2)) to FS data (Figure 1b).

In both healthy and pathological samples, the SS and FS tests revealed a significant predominance of the elastic modulus (*G′*) over the viscous (*G″*), with *G′* being approximately five times greater than *G″*. The shape of the relaxation spectra (*G_i_* substantially constant with λ_i_) confirmed the prevalence of elastic components over viscous, indicating that our samples exhibit rheological behavior like gels [21]. Interestingly, when considering pathological and healthy liver samples together, we found a direct statistical correlation between *G* and *G*_c_ (*r_sp_* = 0.87, *p* < 10^−4^), like that observed in scleroglucan hydrogels [22] (Figure 1d).

**Figure 2:**
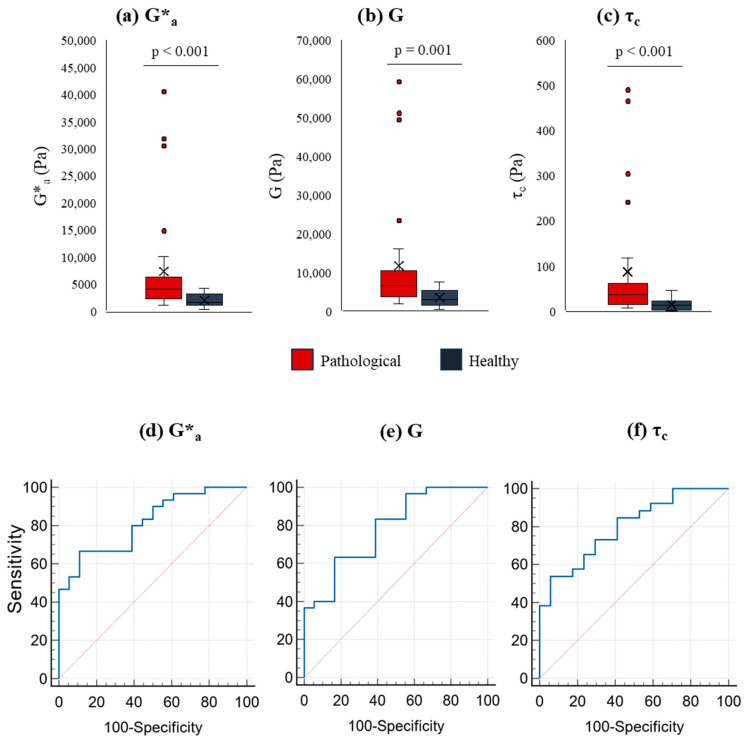
Mean value of the complex modulus $G_{a}^{*}$, shear modulus *G* and critical stress τ_c_ in pathological and normal liver tissues. Boxplots showing the mean value ($G_{a}^{*}$) of the complex modulus G* (**a**), shear modulus *G* (**b**) and critical stress τ_c_ (**c**) for pathological (red) and healthy samples (dark gray). Boxplots visualize data distribution from the first (Q1) to the third quartile (Q3), with the median as a central line and the mean marked by an “×”. Whiskers extend to the smallest and largest values within 1.5 times the interquartile range (Q3–Q1), while outliers beyond this range are shown as individual points. In the case of $G_{a}^{*}$, the average value ± SEM is (7318 ± 1768) Pa for pathological samples and (1991 ± 294) Pa for healthy samples. The average *G* ± SEM for pathological samples is (11,672 ± 2723) Pa and for healthy samples is (3491 ± 536) Pa. The average τ_c_ ± SEM for pathological samples is (87 ± 26) Pa and for healthy samples is (15 ± 3) Pa. Statistical significance was determined by comparing pathological samples with healthy samples: *p* ≤ 0.001. Statistical analyses were conducted using the Mann–Whitney test. (**d**–**f**): area under the ROC curve for $G_{a}^{*}$ (0.817), G (0.785), and τ_c_ (0.799), *p* < 10^−4^.

Considering the prevalence of the elastic properties mentioned above and the significant role played by viscous properties, a comparative rheological analysis of pathological and healthy liver samples was performed. The following parameters were considered: shear modulus *G*, critical stress *τ_c_* (see Equation (5)) and mean complex modulus $G_{a}^{*}$. While G and *τ_c_* are primarily connected to elastic properties, $G_{a}^{*}$ accounts for both elastic and viscous properties (see Equation (2)). Compared to healthy livers, the mean $G_{a}^{*}$, *G*, and *τ_c_* were significantly higher in pathological samples (Figure 2a–c). Additionally, a receiver operating characteristic (ROC) analysis (Figure 2d–f) indicated a high accuracy of $G_{a}^{*}$, *G* and τ_c_ in distinguishing patients from healthy controls (area under the ROC curve of 0.817, 0.785 and 0.799 for $G_{a}^{*}$, *G* and τ_c_, respectively).

**Figure 3:**
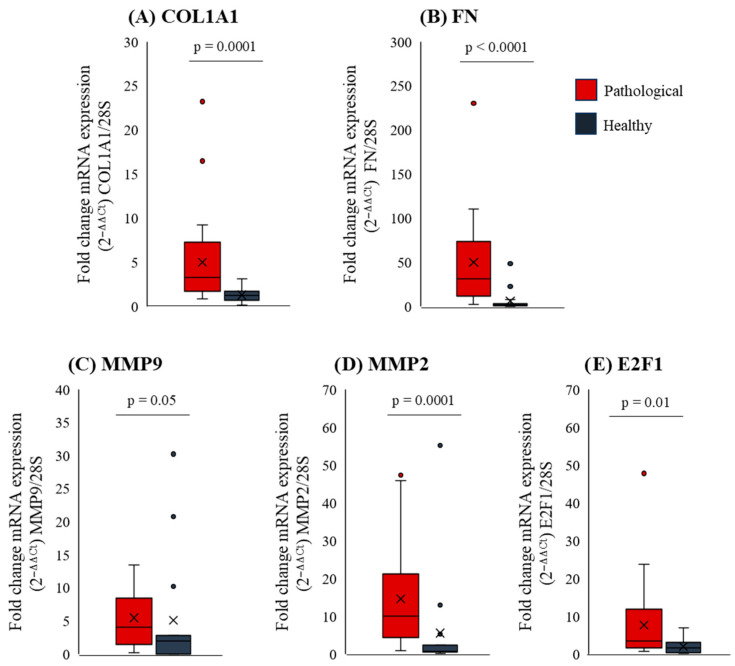
Boxplots showing the Fold change in mRNA expression (2^−ΔΔCt^) of various liver fibrosis related genes normalized to the 28S housekeeping gene. The pro-fibrogenic genes examined are (**A**) collagen type I (COL1A1), (**B**) fibronectin (FN), (**C**,**D**) matrix metalloproteinase 2/9 (MMP-2, MMP-9), and (**E**) E2F1, evaluated in both normal (red) and pathological (dark gray) liver tissues. The boxplots display the distribution of the data from the first (Q1) to the third quartile (Q3), with the median as the central line and the mean marked by an “×”. Whiskers reach the smallest and largest values within 1.5 times the interquartile range (Q3–Q1), while outliers beyond this range appear as individual points. Statistical analyses were conducted using the Mann–Whitney test; *p* = 0.0001 for COL1A1, *p* < 0.0001 for FN, *p* = 0.05 for MMP9, *p* = 0.0001 for MMP2 and *p* = 0.01 for E2F1, when comparing pathological and normal tissue.

Consistent with the increased viscoelastic properties $G_{a}^{*}$ observed by rheology, an increased expression of the pro-fibrogenic proteins collagen type I, fibronectin, E2F1, MMP-2, and MMP-9 was detected (Figure 3A–E), in line with the literature [3,4,5,6].

### 2.3. Rheology vs. Histology

As for elastography, a comparison was made between liver fibrosis determined by histological analysis and rheology. The shear moduli *G*, a crucial rheological parameter for assessing liver elasticity, were obtained from Equations (1) and (2) fitting to FS data (Appendix A.4) and classified into the corresponding fibrosis stages (F0–F4) according to [20], as outlined in the Materials and Methods section. In 17 out of 30 cases, a correlation was observed between histology and rheology in terms of the presence or absence of fibrosis (see Table 1, patients highlighted in green). These findings suggest the potential value of rheology in identifying liver fibrosis.

### 2.4. Low Field Magnetic Nuclear Resonance

**Figure 4:**
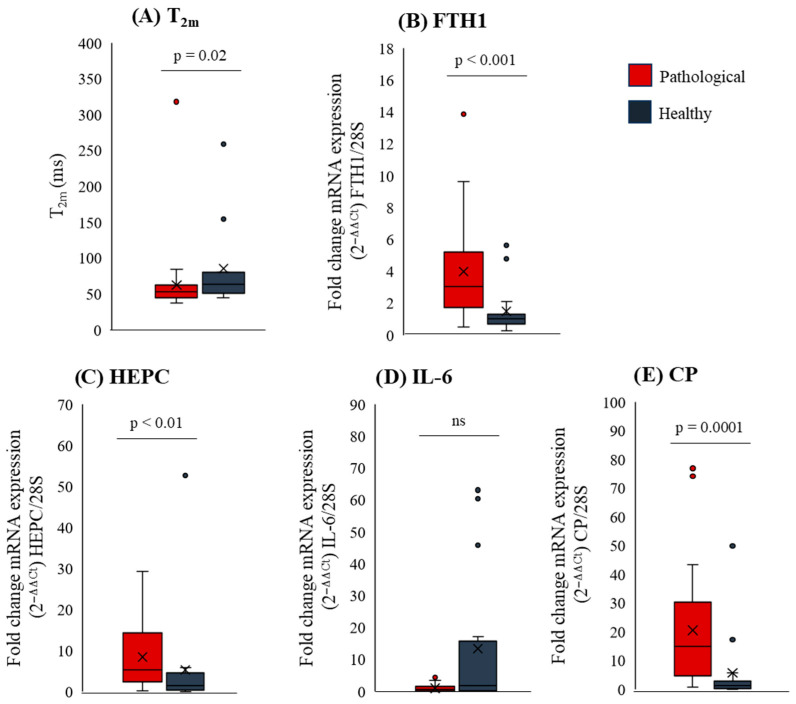
Boxplots showing (**A**) *T*_2m_ and (**B**) ferritin (FTH1); (**C**) hepcidin (HEPC); (**D**) IL-6; (**E**) ceruloplasmin (CP) fold change mRNA expression (2^−ΔΔCt^) normalized to 28S housekeeping gene evaluated in normal (dark gray) and pathological (red) liver tissues. Boxplots visualize data distribution from the first (Q1) to the third quartile (Q3), with the median as a central line and the mean marked by an “×”. Whiskers extend to the smallest and largest values within 1.5 times the interquartile range (Q3–Q1), while outliers beyond this range appear as individual points. Statistical analyses were conducted using the Mann–Whitney test; *p* = 0.02 for *T*_2m_, *p* < 0.001 for FTH1, *p* < 0.01 for HEPC and *p* = 0.0001 for CP, when comparing pathological vs. normal tissue.

Excessive iron in the liver can accelerate disease progression by leading to fibrosis [23]. To evaluate iron levels in the liver, *T*_2m_ measurements were utilized. Due to the ferromagnetic properties of iron that cause rapid relaxation of hydrogen atoms in water molecules, *T*_2m_ is inversely proportional to iron content [18], as depicted in Appendix A.9. In our pathological samples, *T*_2m_ (Figure 4A) was significantly lower compared to healthy controls, indicating increased iron content in pathological livers. This was supported by higher expression of liver ferritin (Figure 4B), the primary iron storage protein [24,25]. Furthermore, elevated hepcidin expression (Figure 4C) further confirms iron accumulation in pathological livers. Hepcidin, primarily produced by liver hepatocytes, increases in response to elevated iron levels [26], inhibiting intestinal iron absorption and preventing iron release from storage/recycling cells in the body. Interestingly, similar levels of IL-6 in pathological tissue compared to controls (Figure 4D) suggest that this inducer of hepcidin synthesis is not a major factor in hepcidin increase [27]. Lastly, our data (Figure 4E) show increased expression of ceruloplasmin, a copper transporter, indicating higher levels of liver copper, a metal that can impact *T*_2m_.

### 2.5. Rheology vs. Low Field Magnetic Nuclear Resonance

**Figure 5:**
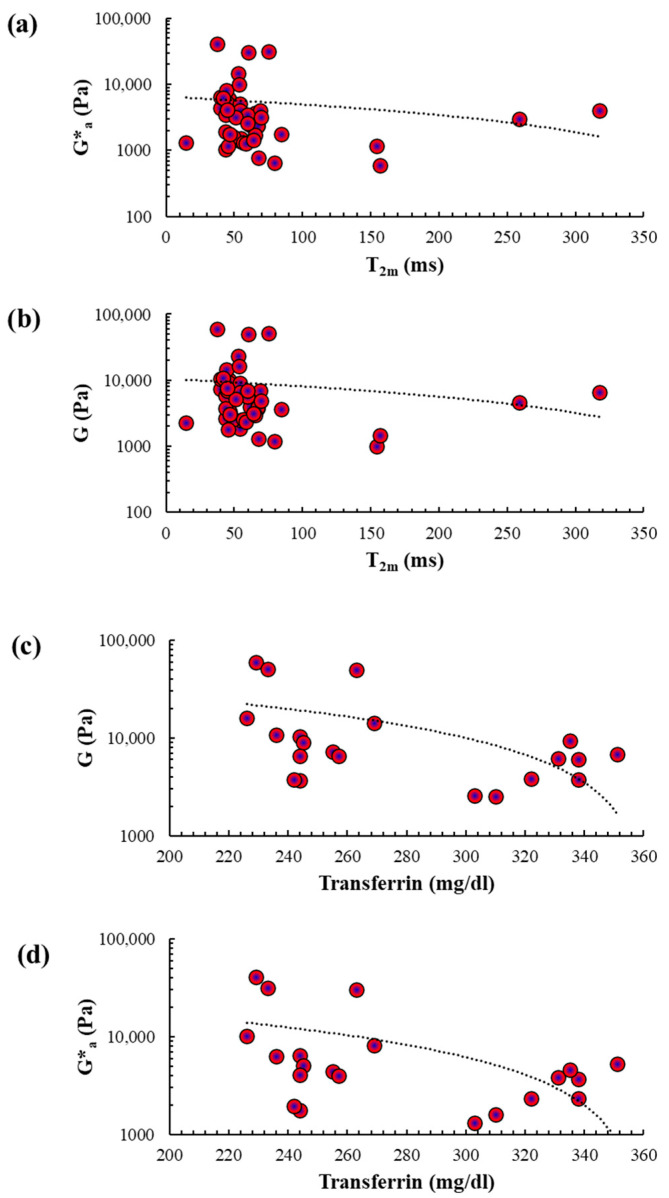
Correlation between *T*_2m_/transferrin and the mean value ($G_{a}^{*}$) of the complex modulus G* or the shear modulus *G* in healthy and pathological liver samples. (**a**) Correlation between *T*_2m_ (σ_T2m_ < 0.05 *T*_2m_) and $G_{a}^{*}$ (σ_G*a_ < 0.15 G*_a_) measured in healthy and pathological liver samples considered together (Spearman correlation with r = −0.32 and *p* < 0.05). (**b**) Correlation between *T*_2m_ and *G* (σ_G_ < 0.05 *G*) measured in healthy and pathological liver samples considered together (Spearman correlation with r = −0.30 and *p* < 0.05). (**c**) Correlation between circulating transferrin (σ_Ctransf_ < 0.15 *C*_transf_) and *G* measured in pathological liver samples (Spearman correlation with r = −0.48 and *p* < 0.05). (**d**) Correlation between transferrin and $G_{a}^{*}$ measured in pathological liver samples (Spearman correlation with r = −0.45 and *p* < 0.05).

We observed intriguing correlations between rheological parameters and *T*_2m_. An inverse correlation was found between *T*_2m_/$G_{a}^{*}$ (Figure 5a) and *T*_2m_/*G* (Figure 5b).

Transferrin, produced by the liver, is the primary transport protein for ferric iron (Fe^3+^) in the bloodstream [24,25]. Moreover, Fe^3+^ bound to transferrin is the main source of iron uptake by hepatocytes. Therefore, we examined the relationship between circulating transferrin levels and LF-NMR/rheological parameters in the subset of patients for whom transferrin blood concentration was available (Appendix A.2). While circulating transferrin concentrations did not correlate with *T*_2m_, an inverse correlation with mean $G_{a}^{*}$ and *G* (Figure 5c,d) was observed.

## 3. Discussion

It is becoming evident that evaluating the rheological properties of the liver is clinically relevant as it can help reveal and monitor liver fibrosis and cirrhosis [28]. In this study, we have undertaken a novel approach by combining rheology and LF-NMR to characterize liver samples from patients with obesity undergoing metabolic and bariatric surgery. The small number of patients included in our pilot study is a limitation. As our cohort primarily consists of patients with minimally fibrotic livers and relatively mild steatotic burden, the representativeness of these patients for the MASLD with more advanced steatosis and fibrosis remains to be determined. It should be noted that patients with advanced fibrosis (F4) are not eligible for bariatric surgery at our hospital, as the reduced liver function associated with severe fibrosis impairs the coagulation system. Another limitation of the study is that the patients come from one center, thus the representativeness of the entire liver fibrotic population needs to be confirmed in a multicenter study. Finally, we do not yet have long-term follow-up data that would be beneficial in fully understanding the prognostic value of our approach.

Previous reports have shown that, in porcine liver tissue [29], the mechanical properties evaluated by rheology are significantly temperature-dependent. While no major modifications are observed up to 20 °C, significant changes occur with increasing temperature. In our study (Appendix A.5, Appendix A.6, Appendix A.7 and Appendix A.8), we found that, at the physiological temperature of 37 °C, the values of the storage modulus (*G′*) and loss modulus (*G″*) increase over time, leading to unacceptable data variability. The reasons for this variability require further investigation, but are likely due to temperature-induced alterations in the liver tissue at 37 °C. In contrast, at 5 °C, the *G′* and *G″* moduli remained stable over time, producing robust and reproducible rheological data. Therefore, the choice of temperature for rheological tests is crucial. Often, in the literature temperature selection is not discussed, or a range of temperatures (20–25 °C) is used [29,30,31,32,33,34], making it difficult to compare results across studies.

Compared to elastography, the direct evaluation of liver tissues by rheology revealed a stronger qualitative correlation with the fibrosis level determined by histology. While the correlation between elastography and histology was only evident in 4 out of 30 liver samples, with rheology this number increased to 17 out of 30 (Table 1). This difference could be attributed, at least in part, to the way rheology and elastography measure tissue elasticity. Indeed, while rheology provides a direct measurement of elasticity, elastography derives tissue elasticity from the measurement of the shear wave velocity. For this purpose, it is important to remember that, although used as synonyms, elasticity (*G*) and stiffness (*K*) do not represent the same physical property. Indeed, *K* (N/m) represents the force (*F*) required to deform a material by a specific amount (*ΔL*), while elasticity *G* (N/m^2^) represents the ability of the sample to return to its original shape after deformation (*ΔL*/*L*_0_). Clearly, they are related (*K* = 3G*A/*L*_0_; *A* = sample cross section, *L*_0_ = original sample length), but represent distinct characteristics of the sample as stiffness depends on both material and geometry, whereas elasticity is a material property related to atomic bond strength. Interestingly, the ROC analysis of the rheological parameters $G_{a}^{*}$, *G* and *τ_c_* (Figure 2d–f) demonstrated high accuracy in distinguishing patients from healthy controls, indicating the diagnostic significance of rheology. Thus, rheology adds value to the diagnosis of fibrosis when liver tissue is available.

The necessity of including a purely elastic component (G_E_ green diamond in Figure 1c) in the generalized Maxwell model to accurately fit the FS data, strongly supports the importance of elasticity (*G′*) in all our samples. It is noteworthy that, in both pathological and healthy samples, the relative importance of viscous and elastic properties is almost the same, as indicated by tan(δ) (=*G″/G′* ≈ 0.22 − δ = dephasing angle). Therefore, the viscous properties cannot be disregarded, as they account for approximately 20% of the elastic properties on average. The relatively high number of Maxwell elements (4–5) required to fit the FS data of both healthy and pathological samples indicates the structural complexity of liver tissue. Despite the similarities in rheological properties between healthy and pathological samples, the latter exhibit higher *G′* and *G″*, resulting in variations in the shear modulus G, which is typically higher in pathological samples. This is reflected in lower *G_i_* values of the relaxation spectrum compared to healthy samples (see Figure 1c).

Liver samples 12 and 17, despite having the same fibrosis degree (1A, as determined by histology, Table 1), exhibit a significant difference in elasticity as assessed by rheology, with *G* values of 23.4 kPa and 3.7 kPa, respectively (Table 1). We believe that this discrepancy may be attributed to the higher lipid content (steatosis), which was 3 in sample 17 and 0 in sample 12. It is plausible that the chemical–physical properties of lipids make the liver tissue softer, leading to reduced elasticity in sample 17. Another explanation could be that the layers used for histological and rheological analysis differ, even though they originate from the same liver sample. This discrepancy may account for the lack of correspondence in 13 out of 30 samples in terms of fibrosis degree between histology and rheology assessment. Therefore, it is recommended that rheological and histological analyses are conducted on liver slices as contiguously as possible.

The rheological analysis of liver tissue allowed the identification of a common feature with gels as reported by Coviello et al. [22], who observed a correlation between the shear modulus *G* and the critical elastic modulus *G*′_c_ in scleroglucan hydrogels. Similarly, when considering our pathological and healthy liver samples together, there is a correlation between *G* and *G′*_c_ (Figure 1d). This correlation suggests that the mechanical properties of liver tissue are closely related to those of hydrogels. The parallelism in rheological behavior between human liver and synthetic hydrogels provides valuable insights for generating in vitro hydrogel models to mimic normal/pathological liver viscoelastic properties. These models can be profitably used to culture liver cells, such as hepatic stellate cells (mainly responsible for liver fibrosis [2]), to study the pathophysiology of human liver fibrosis, as well as the effects of antifibrotic drugs.

For a more comprehensive characterization, the liver samples were also analyzed by LF-NMR to measure *T*_2m_. The reduced value of *T*_2m_ (Figure 4A) in pathological samples indicates an increased iron level as previously observed in a rat model of iron liver accumulation [18]. This is confirmed by the increased expression of ferritin (Figure 4B), a well-known iron storage protein [24], and hepcidin (Figure 4C), both of which are overexpressed in response to elevated iron levels [26]. The fact that the inflammatory cytokine IL-6, an inducer of hepcidin, is not elevated allows us to exclude this cytokine as a cause of hepcidin increase [27]. More generally, the negligible inflammation capable of promoting hepcidin expression, is confirmed by the minimal elevation on average of C reactive protein (Appendix A.2, 6.1 ± 4.4 mg/L, normal value < 5 mg/L). Together, the above molecular data supports the genuine increase of liver iron detected by *T*_2m_. Finally, the histological analysis of iron detection (Perls staining) revealed an increase in iron content in only 2 out of 30 samples (patient number 11 and 19 from Table 1). This indicates that *T*_2m_ can assist histology in detecting iron content. Detecting early iron accumulation in the liver is relevant, as excessive liver iron can lead over time to liver fibrosis, cirrhosis, and eventually hepatocellular carcinoma [23].

The increase of ceruloplasmin (Figure 4E) suggests an increase in copper, a metal which can reduce *T*_2m_. However, the increase has to be modest as none of our patients is affected by Wilson disease, where copper level can significantly increase in the liver. Thus, we believe that the trace copper concentration in the liver (normal range 15–55 μg/g of dried tissue [35] compared to that of iron (normal range 200–2000 μg/g of dried tissue [36]) makes the copper contribution to *T*_2m_ decrease reasonably modest in our patients.

Our data show an inverse correlation between *T*_2m_ and $G_{a}^{*}$/*G* moduli (Figure 5a,b). The easiest interpretation is that iron accumulation (low *T*_2m_) is associated with increased liver elasticity/viscoelasticity. This aligns with the understanding that iron build-up in the liver promotes fibrosis [23], as observed in rats [18]. It is well-established that, when transferrin saturation exceeds 75%, non-transferrin-bound iron (NTBI) starts to accumulate in the liver parenchyma [37]. NTBI then triggers the production of reactive oxygen species (ROS) through the Haber–Weiss reaction. This leads to an elevation in hydroxyl radicals, resulting in phospholipid peroxidation, oxidation of amino acid side chains, and protein fragmentation. These modifications to biological molecules lead to injury and necrosis of hepatocytes, promoting inflammatory events that sustain liver fibrosis. Additionally, NTBI promotes the expression of profibrotic collagen I and TGF-β in HSCs. NTBI also induces the expression of α-SMA, a marker of HSC activation. The effects of NTBI are not limited to HSCs and hepatocytes, as they also affect Kupffer cells, the largest non-parenchymal cell population in the liver. Specifically, NTBI stimulates Kupffer cells to release pro-inflammatory cytokines, along with various proliferative, pro-inflammatory and profibrogenic mediators, with TGF-β being the most significant. TGF-β, in turn, perpetuates HSC activation in a detrimental cycle. Finally, the increase in pro-inflammatory molecules leads to the infiltration of circulating immune cells into the liver tissue, further reinforcing the profibrotic stimuli. Since our patients mostly have mild liver fibrosis (Table 1), we can conclude that the combined use of rheology and LF-NMR can detect liver changes, providing novel possibilities in diagnosis and disease monitoring. Moreover, we wish to emphasize that our approach based on LF-NMR does not aim to replace established methods. Instead, it provides a highly sensitive, non-destructive biophysical approach that is influenced by local magnetic environments. Therefore, it is responsive to variations in tissue iron content.

We also observed an inverse correlation between $G_{a}^{*}$/*G* moduli and circulating transferrin (Figure 5c,d), which is the major transport protein of iron in the blood [24,25]. Since transferrin is produced by the liver, it is plausible that the fibrotic liver gradually loses its functions, including the ability to produce transferrin. This theory is supported by the fact that serum transferrin levels are significantly lower in cirrhotic patients compared to normal subjects [38]. However, our patients are not cirrhotic but have liver fibrosis (Table 1) with little impairment of liver function and circulating transferrin levels within the normal range on average (Appendix A.2). Therefore, our data suggest that the determination of $G_{a}^{*}$/*G* moduli can detect changes in liver functions well before they manifest as a pathological condition. It is worth noting that *T*_2m_ does not correlate with blood transferrin levels, as we believe that it only reflects liver iron content and not the amount of circulating iron. Alternatively, the lack of availability of circulating transferrin for all patients may contribute to the lack of correlation and suggests that further tests are needed to clarify this aspect.

## 4. Conclusions

We have shown that the combined use of rheology/LF-NMR can detect alterations in the liver of patients with obesity, especially in relation to liver viscoelastic properties and iron content. Therefore, rheology and LF-NMR can enhance our understanding of the pathological characteristics of the human liver, aiding in the accurate diagnosis and staging of liver fibrosis.

## 5. Materials and Methods

### 5.1. Rheology

A stress-controlled HAAKE MARS III (Thermo-Scientific, Waltham, MA, USA) rheometer was used, equipped with a 20 mm diameter knurled plate system to prevent slip between the sample and the stationary (lower) and rotating (upper) plates. Gap optimization was determined for each sample by conducting a series of short stress sweep tests (f = 1 Hz; stress range 1–5 Pa) with varying gap distances. The selected gap was the one that maximized the elastic modulus G′ gap distances ranged between 2 and 4 mm. A glass bell, known as a ‘solvent trap’, was used minimize water evaporation from the sample, reducing changes in rheological properties due to water loss. To determine the optimal temperature for rheological analysis, time sweep (TS) measurements were performed for 30 min at two different temperatures (5 °C and 37 °C) to evaluate the time variation of G′ and G″ (f = 1 Hz, τ = 5 Pa). This test was repeated twice with two randomly selected samples (sample 19: Appendix A.5 and Appendix A.6. Sample 20: Appendix A.7 and Appendix A.8). At 37 °C, the values of G′ and G″ began to increase around 300 s, indicating changes in the rheological properties of the liver tissue. In contrast, at 5 °C, the G′ and G″ moduli remained stable, suggesting no significant changes in the tissue’s rheological properties or structure. Based on these results, 5 °C was selected as the temperature for the analyses in this study. Freshly isolated human liver biopsies were kept on ice to prevent sample deterioration until experimental analysis. The liver biopsies were then sliced to about 3 mm thickness and gently placed between the upper and lower plates of the rheometer for measurement. Rheology tests were conducted in triplicate and in a blinded manner.

Two types of oscillatory rheological tests were conducted: the stress/strain weep (SS) test and the frequency (FS) test. For the SS measurement, the following parameters were used: shear stress *τ* = 1–1000 Pa, frequency f = 1 Hz. In the FS measurement, the parameters used were shear stress *τ* = 1 or 5 Pa (based on the width of the sample’s linear viscoelastic region determined by SS test), and frequency f = 0.1–10 Hz. The frequency sweep data were analyzed using the generalized Maxwell model:

(1) $$G^{\prime }\;=\;G_{E}+\sum_{i=1}^{n_{R}}G_{i}\frac{{\left(\lambda_{i}\omega \right)}^{2}}{1+{\left(\lambda_{i}\omega \right)}^{2}}\;\;\;\;\;\;\;\;\;\eta_{i}\;=\;\lambda_{i}G_{i}$$

(2) $$G^{\prime \prime }=\sum_{i=1}^{n_{R}}G_{i}\frac{\left(\lambda_{i}\omega \right)}{1+{\left(\lambda_{i}\omega \right)}^{2}}$$

where ω is the solicitation frequency (=2π*f*; *f* is the solicitation frequency), *n*_R_ is the number of Maxwell elements considered, whereas *g*_E_ (elastic constant of the purely elastic Maxwell element), *G*_i_ (ith elastic constant) and λ_i_ (ith relaxation time) represent model fitting parameters. Model fitting was performed assuming that λi was scaled by a factor of 10 (λ_i+1_ = 10λ_i_) [39]. The *n*_R_ determination was performed according to a statistical procedure, in order to minimize the product *N*χ^2^, where *N* indicates the number of fitting parameters and χ^2^ is the sum of the squared errors [40]. Three parameters were considered to represent the rheological properties of each sample: the shear modulus *G*, the average complex modulus $G_{a}^{*}$, and the critical stress τ_c_. While *G* was evaluated as the sum of all *g*_i_ (*G* = Σ*G*_i_) [21], the average complex modulus $G_{a}^{*}$ was estimated by averaging the complex modulus:

(3) $$G^{*}\;=\;\sqrt[{2}]{{\left(G^{\prime }\right)}^{2}+{\left(G^{\prime \prime }\right)}^{2}}$$

across the entire pulsation (ω) range considered. Finally, γ_c_ (limit of the linear viscoelastic regime over which sample nanostructure undergoes damaging) was evaluated by fitting the Soskey–Winter equation [41] to the experimental strain sweep data:

(4) $$G^{\prime }=\frac{G_{0}^{\prime }}{1+{\left(b\gamma \right)}^{n}}$$

where $G_{0}^{\prime }$ is the *G′* value occurring for a vanishing deformation (γ ≈ 0). *b* and *n* are two fitting parameters. The value of γ_c_ is calculated by Equation (4), setting $G^{\prime }\;=\;0.95\;\times \;G_{0}^{\prime }$ [42]. The corresponding value of the critical stress τ_c_ was evaluated according to

(5) $$\tau_{c}=G_{c}^{\prime }{\;\gamma }_{c}$$

where *G′*_c_ represents the values of the elastic (*G′*) modulus values occurring at γ_c_ (i.e., the critical *G′* value). A glass bell known as a ‘solvent trap’ was used to prevent water evaporation from the sample, minimizing changes in rheological properties due to water loss. To determine the optimal temperature for rheological analysis, TS measurements were conducted for 30 min at two different temperatures (5 °C and 37 °C). The oscillatory time sweep was performed at constant maximum stress (5 Pa) and frequency of 1 Hz. This test was repeated twice with two randomly selected samples (sample 19: Appendix A.5 and Appendix A.6. Sample 20: Appendix A.7 and Appendix A.8). At 37 °C, the values of the storage modulus (*G′*) and loss modulus (*G″*) began to increase at around 300 s, indicating changes in the rheological properties of the liver tissue. In contrast, at 5 °C, the *G′* and *G″* moduli remained stable, suggesting no significant changes in the tissue’s rheological properties or structure. Based on these results, 5 °C was selected as the temperature for the analyses in this study. Rheology tests were conducted in a blinded manner.

To analyse the relationship between Kleiner–Brunt fibrosis and steatosis stages and the rheological data, the following correlation was utilized [20]: F0: 1–6 kPa, F1(1A, 1B, 1C): 6.1–7 kPa, F2: 7.1–9 kPa, F3: 9.1–10.3 kPa, F4: ≥10.4 kPa.

### 5.2. LF-NMR

The extinction of the transversal component (*M*_xy_) of the magnetization vector (*M*) (transversal relaxation) was recorded at 37 °C by a Bruker Minispec MQ20 (static magnetic field *B*_0_ = 0.47 T, 20 MHz, Billerica, MA, USA) resorting to the CPMG sequence (Carr–Purcell–Meiboom–Gill), 23, with a sequence of {90°[-τ-180°–τ(echo)]_k_-*T*_R_}, an 8.36 μs wide 90°pulse, τ = 250 μs and *T_R_* (recycle delay) equal to 10 s. Each relaxation experiment was repeated 36 times (four scans for each of the 9 repetitions performed on the same sample) (See Appendix A.1 for further experimental details). Freshly isolated human liver biopsies were kept on ice to prevent sample deterioration until experimental analysis. Small cylindrical samples (with a diameter of less than <8 mm and a height of less than <1 cm) were obtained from liver biopsies and placed inside a glass sample holder, which was immediately sealed with a plastic cap. LF-NMR tests were conducted in a blinded manner.

The determination of *T*_2m_ was achieved by fitting the experimental relaxation data to a sum of exponential terms each characterized by a different time decay constant (*T*_2i_) and weight (*A*_2*i*_) [43]:

(6) $$I(t)\;=\;\sum_{i=1}^{m}A_{2i}e^{\left(-t/T_{2i}\right)}$$

where *I*(*t*) is the dimensionless signal amplitude that becomes negligible at the end of the relaxation process. The number, *m*, of exponential decays appearing in Equation (6) was determined by a statistical [40] procedure based on the minimization of the product (2*m*χ^2^) where *χ*^2^ is the sum of the squared errors and 2*m* is the number of model fitting parameters. The average spin–spin relaxation time *T*_2*m*_ can be evaluated by the following equation:

(7) $$T_{2m}=\sum_{i=1}^{m}A_{2i}T_{2i}/\sum_{i=1}^{m}A_{2i}\;\;\;\;\;\;A_{2i\%}=100\;A_{2i}/\sum_{i=1}^{m}A_{2i}$$

The *m* couples (*T*_2*i*_, *A*_2*i%*_) represent the discrete relaxation time distribution referring to the transversal and longitudinal relaxation.

To investigate the relationship between iron concentration and *T*_2m_, we prepared aqueous solutions with varying concentrations of iron(II) sulfate (FeSO_4_) and measured *T*_2m_ at 25 °C for each solution. The results, shown in Appendix A.9, indicate that, as the iron concentration increases, *T*_2m_ decreases, although not in a linear manner.

### 5.3. Liver Sample Collection and Preparation

Thirty consecutive patients who underwent metabolic and bariatric surgery at the University Hospital of Cattinara, Trieste, Italy, were included in this study. The study was approved by the Ethics Committee of the University of Trieste (permission number 126, 1 December 2022) and all procedures were performed in compliance with relevant laws and the guidelines of the University of Trieste. Moreover, the research has been carried out in accordance with the Declaration of Helsinki. Written informed consent was obtained from all patients. Metabolic and bariatric surgery, also known as weight-loss surgery, involves various procedures that modify the digestive system to aid in weight loss and improve weight-related co-morbidities. The clinical and haemato-chemical characteristics of the patients are outlined in Table 1 and Appendix A.2. All participants were obese patients. Obesity was classified according to [44] as follows: Class I (Mild) Obesity with a BMI between 30.0 and 34.9, Class II (Moderate) Obesity with a BMI between 35.0 and 39.9, Class III (Severe or Morbid) Obesity with a BMI equal to or greater than 40.0 The diagnostic criteria for MASLD were based on those reported in [45]. Surgically removed human liver tissues were immediately transported to the laboratory and stored on ice. Additionally, 18 liver samples were collected from patients who underwent extensive hepatic resection due to metastatic cancer to serve as controls. These liver samples were taken from areas unaffected by metastases. Histological analysis was conducted for both groups using standard procedures and classified according to the Kleiner–Brunt fibrosis and steatosis stages [19]. Fibrosis was categorized into eight levels (0, 1, 1A, 1B, 1C, 2, 3, and 4) while steatosis was classified into four levels based on the percentage of hepatocytes containing fat droplets (S0: <5%, S1: 5–33%, S2: 34–66%, S3: >66%). The histological analysis was performed on formalin-fixed, paraffin-embedded liver tissue sections (3–4 μm thick), stained with hematoxylin and eosin (H and E) for general morphology and Masson’s trichrome staining to evaluate fibrosis (representative examples are reported in Appendix A.10). An experienced pathologist, blinded to the rheological and LF-NMR data, conducted the evaluation. In cases of uncertainty, slides were reviewed jointly to reach a consensus diagnosis. Hepatic iron overload was assessed using semi-quantitative histological grading such as Perls staining, which reflects the biochemical quantification of hepatic iron concentration. It is important to note that our patients do not have hemochromatosis or transfusional iron overload. Perl staining was assessed in both hepatocytes and Kupfer cells with positive samples defined as those showing Perls staining in at least one cellular type and negative samples showing no positivity in either hepatocytes or Kupffer cells.

For rheology and LF-MNR investigations, live slices of approximately 1 cm^2^ with a thickness of about 3 mm were prepared under a laminar flow hood. Subsequently, a rheology test was conducted, followed by LF-NMR examination. Samples intended for molecular analyses were also prepared and flash-frozen in liquid nitrogen, then stored at −80 °C until RNA extraction.

### 5.4. Total RNA Extraction from Liver Tissue Samples

Total RNA was extracted following tissue lysis and homogenization. Approximately 30 mg of liver samples, kept on ice, were homogenized in 800 µL of TRIzol Reagent (Invitrogen, ThermoFisher Scientific, Waltham, MA, USA) using an Ultra-Turrax T 25 basic homogenizer (IKA). The resulting lysates were centrifuged at 12.000 rcf for 10 min at 4 °C, and the clear supernatant was transferred to a new tube. After a 5-min incubation at room temperature to aid the dissociation of nucleoprotein complexes, 160 µL of chloroform was added. Samples were vigorously mixed by shaking and then incubated for 2–3 min at room temperature. Phase separation was achieved by centrifugation at 12.000 rcf for 15 min at 4 °C. The colorless upper aqueous phase, containing total RNA, was transferred to a new tube for purification using the RNeasy Mini Kit (Qiagen, Venlo, The Netherlands), following the manufacturer’s instructions. RNA was eluted in 30 µL of RNase-free water through centrifugation. RNA concentration and purity were determined using a NanoDrop ND-100 spectrophotometer (Thermo Scientific, Wilmington, DE, USA).

### 5.5. Quantitative Real Time PCR

One μg of each RNA sample was reverse transcribed into cDNA using TaqMan Reverse Transcription Reagents (ThermoFisher Scientific, Waltham, MA, USA). The master mix for reverse transcription was prepared according to the manufacturer’s instructions using random hexamers. Subsequently, the cDNA was used as a template for quantitative-PCR using PowerUp SYBR Green Master Mix (Applied Biosystems Waltham, MA, USA) on the StepOnePlus Real-Time PCR System (Applied Biosystems). The primer sequences (Eurofins/ThermoFisher Scientific Waltham, MA, USA) were as it follows: collagen type I (COL1A1) (FW 5′-TCG TCA CAG ATC ACG TCA TCG-3′ and RV 5′-AAT CAC CTG CGT ACA GAA CGG-3′), fibronectin (FN) (FW 5′-ACC AAC CTA CGG ATG ACT CG-3′ and RV 5′-GCT CAT CAT CTG GCC ATT TT-3′), MMP-9 (FW 5′-CTG GAG GTT CGA CGT GAA G-3′ and RV 5′-TCC TGG CAG AAA TAG GCT TTC-3′), MMP-2 (FW 5′-CCA TGA TGG AGA GGC AGA CA-3′ and RV 5′-TCC GTC CTT ACC GTC AAA GG-3′), E2F1 (FW 5′-CCA GGA AAA GGT GTG AAA TC-3′ and RV 5′-AAG CGC TTG GTG GTC AGA TT-3′), ferritin (FTH1) (FW 5′-TGA AGC TGC AGA ACC AAC GAG G-3′ and RV 5′-GCA CAC TCC ATT GCA TTC AGC C-3′), hepcidin (HEPC) (FW 5′-CTG ACC AGT GGC TCT GTT TTC C-3′ and RV 5′-AAG TGG GTG TCT CGC CTC CTT C-3′), IL-6 (FW- 5′-GCT GAA AAA GAT GGA TGC TTC-3′ and RV 5′-ACT CCA AAA GAC CAG TGA TG-3′) and ceruloplasmin (CP) (FW 5′-CCC TCA AAC AAG TCT TAC GCT CC-3′ and RV 5′-CCA GGT AGA AGG TGG AAT CCT C-3′). The expression level of the target genes was normalized to the housekeeping gene 28S, (FW 5′-TGG GAA TGC AGC CCA AAG-3′ and RV 5′-CCT TAC GGT ACT TGT TGA CTA TCG-3′) and analyzed using the 2^*−ΔΔCt*^ method.

### 5.6. Statistical Analysis

Statistical analyses were conducted using GraphPad InStat. Data are presented as mean ± standard error of the mean (SEM). Normality of distribution was assessed using the Kolmogorov–Smirnov test. Depending on data distribution, comparisons between two groups were performed using either the unpaired Student’s *t*-test (for normally distributed variables) or the Mann–Whitney U test (for non-normally distributed variables). *p*-values < 0.05 were considered statistically significant. Correlations between variables were evaluated using Pearson’s or Spearman’s coefficients, depending on data distribution. The receiver operating characteristic (ROC) analysis was performed by MedCalc^®^ Statistical Software version 23.3.1 (MedCalc Software Ltd., Ostend, Belgium; http://www.medcalc.org; 2024, accessed on the 1 June 2025).

## Data Availability

All data are available upon request to the corresponding authors.

## Abbreviations

The following abbreviations are used in this manuscript:

ALT	Alanine amino transferase
AST	Aspartate amino transferase
B	bilirubin
CP	ceruloplasmin
COL1A1	collagen type I
CRP	C reactive protein
ECM	extracellular matrix
FN	fibronectin
FS	frequency sweep
FTH1	ferritin
γGT	Gamma-glutamyl transferase
HEPC	hepcidin
IL-6	interleukin 6
LF-NMR	Low Field-Nuclear Magnetic Resonance
MASLD	metabolic dysfunction-associated steatotic liver disease
MMPs	matrix metalloproteinases
SS	stress sweep
Trf	transferrin

## Appendix A

### Appendix A.1. LF-NMR

LF-NMR relies on the ability of hydrogen atoms dipole to react to the perturbation of an external constant magnetic field B_0_ (conventionally directed along the z-axis, i.e., the longitudinal direction), where they are embedded. Essentially, the application of a radio frequency pulse B_1_, lying in the x–y plane perpendicular to B_0_, induces dipole orientation in the x–y plane. After the removal of B_1_, the dipoles tend to return to their initial alignment with B_0_ (relaxation). The relaxation process can be described by the average spin-spin relaxation time, T_2m_, that measures the extinction of M_xy_, the x-y (or transversal) component of the magnetization vector (M). M_xy_ extinction was measured at 37 °C using a Bruker Minispec MQ20 (static magnetic field B0 = 0.47 T, 20 MHz, Rheinstetten, Germany), a bench instrument that can be easily hosted in a hospital laboratory. It does not require particular safety requirements due to the low magnetic field involved. Each sample was poured into a glass tube (internal diameter 0.008 m) sealed with a proper plastic top immediately after insertion. The glass tube was then kept at 37 °C for approximately ten minutes before measurement. Finally, it was quickly inserted into the MQ20 sample holder positioned just above the magnetic field.

M_xy_ extinction was recorded at 37 °C by a Bruker Minispec MQ20 (static magnetic field B_0_ = 0.47 T, 20 MHz, Germany) resorting to the CPMG sequence (Carr-Purcell-Meiboom-Gill) [46] with a sequence of {90°[-τ-180°–τ(echo)]_k_-T_R_}, an 8.36 μs wide 90°pulse, τ = 250 μs and T_R_ (recycle delay) equal to 10 s. The variable k represents the number of experimental echoes and is approximately related to the experimental test duration, T_d_, by T_d_ = (2τ)*k = (2 τ) k’*(1 + A), where k’ is the number of recorded echoes and A is the number of not recorded echoes. Therefore, k = k’*(1 + A). The trial-and-error procedure used to select k’ (≤1000) and A (≤21) ensured that, at the end of the experiment (t = T_d_), the M_xy_ intensity (FID or I_s_(t)) was approximately 2% of the initial intensity. As a result, the time interval (T_d_/k’) for data acquisition is equal to 2τ*(1 + A) and may vary from sample to sample due to the different k’ and A values chosen to achieve the desired T_d_. Each relaxation experiment, consisting of k’ points, was repeated 36 times (four scans for each of the 9 repetitions performed on the same sample).

The LF-NMR equipment was validated every day according to an internal procedure named Daily Check that uses an internal standard. This serves to automatically recalibrate the instrument’s parameters and check the performance of the system, such as signal strength or temperature stability. The 36 repetitions performed on each sample proved the high reproducibility of the measurement (signal intensity). Indeed, the highest values of standard deviation and coefficient of variation were 1.77 and 0.038, respectively. T_2m_ measurements were performed in a blinded fashion.

### Appendix A.2. Biochemical Liver Function Tests of Patients and Comorbidities

**Pat. N.**	**AST** **(U/L)**	**ALT** **(U/L)**	**γGT** **(U/L)**	**Total B (mmol/L)**	**Direct B** **(mmol/L)**	**Albumin (g/dL)**	**Trf** **(mg/dL)**	**CRP** **(mg/L)**	**Comorbidities**
1	14	16	44	0.4	0.08	4.38	338	7.2	DM
2	22	25	16	0.79	0.16	4.27	255	6.7	IR
3	29	28	66	0.72	0.13	4.72		16.3	IR
4	58	109	56	0.54	0.1	4.69	244		AH, DM
5	25	23	15	0.52	0.08	4.17	331	4.8	IR, load-bearing arthropathies
6	19	31	70	0.58	0.09	4.63			AH, DY, OSAS
7	23	23	20	0.31	0.06	4.27		7.6	DM, AH, OSAS
8	19	29	42	0.33	0.06	4.36			AH, OSAS
9	15	16	25	0.67	0.12	3.92		10.3	AH
10	22	27	25	0.49	0.09	4.42	335	0.9	DM, AH
11	19	9	13	0.51	0.08	4.1		1.8	AH, HC
12	22	27	44	0.74	0.13	4.56		3.2	IR
13	27	36	39	0.8	0.17	4.54	229		DM, AH, HC
14	26	31	24	0.99	0.18	4.52		1.8	DM, AH, HC
15	23	13	25	0.65	0.11	4.08	351	3.8	IR
16	18	35	27	0.58	0.13	4.15	245	3.6	DM, OSAS
17	40	115	98	0.58	0.09	4.25	244	14.3	IR
18	22	25	41	0.95	0.15	3.98	257	4.8	IR, OSAS
19	20	23	17	2.34	0.39	4.07	242	1.3	IR, OSAS
20	24	33	25	0.33	0.06	4.27		12.4	DM, AH
21	23	25	18	0.52	0.08	4.39	338	5.3	AH
22	17	13	24	0.73	0.13	4	322	12.4	DM
23	20	23	26	0.95	0.18	4.67	310	3.2	DM, DY
24	23	34	34	0.6	0.12	4.52	303		IR
25	42	48	43	0.93	0.21	4.54	244	0.6	DM
26	18	27	17	0.5	0.1	3.63	263	5.6	DM, AH
27		18	16	0.58	0.12	3.87	269	11.8	IR, load-bearing arthropathies
28	22	38	217	0.59	0.14	4.14	226	6.5	DM, AH, DY
29	21	16	22	1.26	0.27	4.63	236	3	DM, AH, OSAS
30	38	74	41	0.92	0.17	4.52	233	3.5	IR, load-bearing arthropathies
AST: Aspartate amino transferase; ALT: Alanine amino transferase; γGT: Gamma-glutamyl transferase; B: bilirubin; Trf: transferrin; C reactive protein: CRP; DM: diabetes mellitus; IR: insulin resistance; AH: arterial hypertension; DY: dyslipidemia; HC: hypercholesterolemia; OSAS: Obstructive Sleep Apnea Syndrome.									

### Appendix A.3. Results of SS Analysis

Data are reported for the healthy (black) and pathological (red) liver samples based on the Soskey–Winter equation (Equation (3)). In this equation, γ_c_ and τ_c_ present the critical deformation and shear stress, respectively, while G′_c_ = τ_c_/γ_c_. G_0′_, b, and n are Equation (3) fitting parameters. The statistical reliability of the best fitting of Equation (3) was always confirmed by the F-test score.

Results of SS Analysis.

**Sample**	**γ_c_ (-)**	***G′*****_c_** **(Pa)**	**τ_c_ (Pa)**	***G′*****_0_** **(Pa)**	***b*** **(-)**	***n*** **(-)**
**1**	-	-	-	-	-	-
**2**	-	-	-	-	-	-
**3**	-	-	-	-	-	-
**4**	-	-	-	-	-	-
**5**	0.0065	5532	36.2	6367	9.33	1.05
**6**	0.0058	20,355	117.8	20,827	56.72	2.64
**7**	0.0149	946	14.1	1123	0.82	0.67
**8**	0.0027	10,553	28.3	10,974	50.12	1.47
**9**	0.0058	2471	14.3	2581	39.08	1.98
**10**	0.0117	4968	58.3	5266	8.27	1.26
**11**	0.0104	2230	23.2	2463	5.81	1.05
**12**	0.0031	14,712	45.3	15,638	5.41	0.72
**13**	0.0043	71,221	304.0	80,343	38.39	1.63
**14**	0.0069	2590	17.9	2597	14.48	1.28
**15**	0.0167	14,472	241.4	18,807	7.62	1.43
**16**	0.0065	8884	58.0	9279	29.69	1.79
**17**	0.0050	3578	17.8	4317	17.49	1.21
**18**	0.0143	34,269	489.9	35,806	18.41	2.21
**19**	0.0098	3726	36.4	3236	7.51	1.13
**20**	0.0066	3399	22.6	3256	6.29	0.93
**21**	0.0037	1818	6.8	2055	2.13	0.61
**22**	0.0052	2696	14.0	3085	3.54	0.74
**23**	0.0057	1251	7.1	1378	1.93	0.65
**24**	0.0106	1098	11.6	1270	2.29	0.79
**25**	0.0033	3382	11.0	3684	2.77	0.63
**26**	0.0020	29,133	57.5	31,633	6.37	0.67
**27**	0.0078	7374	57.7	8049	4.31	0.87
**28**	0.0089	8200	73.2	7677	2.91	0.81
**29**	0.0068	5297	36.0	6045	3.56	0.79
**30**	0.0201	23,086	465.1	28,820	4.37	1.21
**1**	0.0085	1942	16.5	1795	3.68	0.85
**2**	0.0161	1854	29.9	1738	6.14	1.27
**3**	0.0051	250	1.3	285	1.23	0.58
**4**	0.0180	698	12.5	702	1.17	0.76
**5**	-	-	-	-	-	-
**6**	0.0184	465	8.6	722	1.12	0.76
**7**	0.0001	2363	0.2	2659	2.44	0.35
**8**	0.0005	1928	1.1	1301	1.55	0.42
**9**	0.0127	1383	17.5	2158	0.63	0.61
**10**	0.0126	3652	45.9	3278	1.89	0.79
**11**	0.0184	1363	25.1	1435	2.24	0.92
**12**	0.0203	1614	32.8	1699	1.89	0.90
**13**	0.0121	1664	20.2	1752	1.97	0.79
**14**	0.0038	2139	8.1	2252	0.93	0.52
**15**	0.0091	1264	11.4	1330	2.66	0.79
**16**	0.0063	1950	12.3	2052	1.03	0.58
**17**	0.0028	883	2.5	2660	3.93	1.12
**18**	0.0007	3286	2.3	3459	1.18	0.41

### Appendix A.4. Results of FS Analysis

Data are reported for the FS analysis on healthy (in black) and pathological (in red) liver samples. *G* (=$\sum_{i}G_{i}$) is the shear modulus while *G*_0_, *G*_1_, *G*_2_, *G*_3_, *G*_4_ and λ_1_ are the fitting parameters of the generalized Maxwell model (Equations (1) and (2)). *E*_%_ expresses the ratio between the purely elastic component (*G*_E_) of the generalized Maxwell model (Equations (1) and (2)) and the shear modulus *G* (=$\sum_{i}G_{i}$). *G*^*^_a_ represents the complex modulus averaged over the entire range of solicitation pulsation (ω) considered in FS test. The statistical reliability of Equations (1) and (2) best fitting was always confirmed by the F-test score.

Results of FS Analysis.

**Sample**	**λ_1_ (s)**	***G*****_E_** **(Pa)**	***G*****_1_** **(Pa)**	***G*****_2_** **(Pa)**	***G*****_3_** **(Pa)**	***G*****_4_** **(Pa)**	***G*** **(Pa)**	***E*** **_%_**	***G*****^*^****_a_** **(Pa)**
**1**	0.0153	805.8	1735.9	848.3	805.1	1885.9	6081.1	13.3	3719
**2**	0.0090	2518.5	1964.6	954.4	806.3	1059.9	7303.8	34.5	4392
**3**	0.0181	612.2	1740.9	848.1	725.0	1846.1	5772.3	10.6	3491
**4**	0.0164	3386.8	2562.1	1488.6	1800.2	1116.6	10,354.3	32.7	6450
**5**	0.0205	1298.0	1746.2	897.7	742.5	1509.6	6194.0	21.0	3838
**6**	0.0162	2747.2	2954.9	1555.2	1451.0	1500.3	10,208.6	26.9	6098
**7**	0.0234	8.0	1417.8	647.2	597.6	-	2670.7	0.3	1046
**8**	0.0150	1864.6	2042.1	1022.1	935.0	1088.6	6952.3	26.8	4060
**9**	0.2517	61.0	646.0	73.0	1096.3	-	1876.2	3.2	1420
**10**	0.0067	2002.1	3231.5	1451.8	1136.8	1465.2	9287.5	21.6	4571
**11**	0.0103	566.4	1341.5	432.4	388.8	615.3	3344.4	16.9	4571
**12**	0.0065	6166.3	5897.8	2930.2	3529.6	4895.8	23,419.9	26.3	14,791
**13**	0.0142	27,123.7	13,146.7	9158.8	9884.5	-	59,313.7	45.7	40,532
**14**	0.0267	59.9	817.4	393.4	414.5	837.4	2522.6	2.4	1508
**15**	0.0539	3372.6	1747.6	666.3	1090.3	-	6876.9	49.0	5264
**16**	0.0102	2123.4	2717.7	1394.8	1184.2	1516.6	8936.6	23.8	5115
**17**	0.0180	1059.4	1165.3	498.3	430.0	501.9	3654.9	29.0	1757
**18**	0.0118	1843.8	1705.6	973.9	814.9	1260.9	6599.1	27.9	4004
**19**	0.0113	762.0	1295.3	614.8	480.3	596.7	3749.0	20.3	1947
**20**	0.0175	1704.9	1261.5	584.5	465.3	-	4016.2	42.5	2458
**21**	0.0189	1325.8	1191.5	595.1	337.6	284.4	3734.3	35.5	2342
**22**	0.0253	1081.4	1175.3	615.8	469.3	503.6	3845.3	28.1	2349
**23**	0.0297	745.0	841.3	408.8	301.6	249.9	2546.6	29.3	1602
**24**	0.0100	683.5	953.7	340.9	324.7	268.7	2571.5	26.6	1318
**25**	0.0156	2374.5	2015.5	901.9	722.6	570.1	6584.6	36.1	4062
**26**	0.0131	13,782.0	11,736.1	7862.9	5009.3	10,980.8	49,371.1	27.9	30,491
**27**	0.0139	4102.2	4421.1	1594.7	2326.7	1888.8	14,333.4	28.6	8159
**28**	0.0137	5414.6	4463.7	1933.9	2580.7	1784.1	16,177.0	33.5	10,104
**29**	0.0143	3599.6	3291.9	1550.6	1258.2	1045.2	10,745.5	33.5	6286
**30**	0.0145	14,435.1	12,924.2	7194.6	8462.7	8108.2	51,124.8	28.2	31,785
**1**	0.0434	378.3	325.8	146.7	159.9	-	1010.7	37.4	1172
**2**	0.0436	732.6	531.8	242.5	275.6	-	1782.5	41.1	1160
**3**	0.0949	108.4	138.9	67.3	66.5	-	381.1	28.4	256
**4**	0.0383	291.9	452.1	194.6	135.7	128.4	1202.7	24.3	648
**5**	0.0093	324.2	594.8	251.2	163.0	135.6	1468.8	22.1	594
**6**	0.0304	395.0	483.5	195.0	153.2	82.5	1309.2	30.2	782
**7**	0.0131	1803.1	1812.8	854.4	742.3	486.7	5699.2	31.6	3468
**8**	0.0301	622.9	868.0	383.8	295.8	208.2	2378.6	26.2	1271
**9**	0.0218	1385.7	1655.3	790.1	637.6	442.4	4911.1	28.2	3161
**10**	0.0197	1656.3	2302.7	1179.8	1121.7	789.0	7049.4	23.5	4059
**11**	0.0677	800.6	793.1	346.3	316.3	-	2256.2	35.5	1339
**12**	0.0654	986.3	1039.9	519.6	405.5	-	2951.4	33.4	1714
**13**	0.0610	974.6	1086.1	565.3	440.9	-	3066.8	31.8	1784
**14**	0.0230	1206.7	1342.2	778.4	1229.8	-	4557.1	26.5	2995
**15**	0.0074	675.8	1290.6	611.9	333.2	248.7	3160.1	21.4	1433
**16**	0.0180	1885.5	1865.8	675.2	722.7	-	5149.2	36.6	3191
**17**	0.0074	975.8	3067.2	1352.0	909.8	653.3	6958.1	14.0	2615
**18**	0.0116	2072.6	2588.0	1145.6	1028.9	711.3	7546.3	27.5	4198

### Appendix A.10. Representative Histological Images

(1) *HE (hematoxylin and eosin)*: diffuse macro-vesicular steatosis, extensively involving the hepatic lobule.

(2) *Masson’s trichrome stain*: demonstrates preserved lobular architecture with no significant collagen deposition, consistent with fibrosis stage F0.

(3) *Perls staining*: no evidence of iron accumulation within the hepatic parenchyma.

(4) *HE (hematoxylin and eosin):* mild macro-vesicular steatosis, predominantly distributed in zone 3 (centrilobular region), without additional histological abnormalities.

(5) *Masson’s trichrome stain:* preserved lobular architecture with no significant collagen deposition, consistent with fibrosis stage F0.

(6) *Perls staining:* iron deposition within hepatocytes, with minimal iron accumulation also observed in Kupffer cells (representative areas indicated by white ellipses).

(7) *HE (hematoxylin and eosin)*: hepatic steatosis associated with a moderate inflammatory infiltrate within enlarged portal tracts.

(8) *Masson’s trichrome stain*: expanded portal areas with formation of fibrous septa and marked thickening of the central vein wall, consistent with fibrosis stage F2.

(9) *Perls staining:* no evidence of iron accumulation within the hepatic parenchyma.

Scalebar 200 μm.
